# Impact of concomitant corticosteroid use on adverse events in ulcerative colitis clinical trials

**DOI:** 10.1093/ecco-jcc/jjaf145

**Published:** 2025-08-05

**Authors:** Hasan Hamam, Parambir S Dulai, John K Marshall, Christopher Ma, Vipul Jairath, Walter Reinisch, Neeraj Narula

**Affiliations:** Department of Medicine (Division of Gastroenterology) and Farncombe Family Digestive Health Research Institute, McMaster University, Hamilton, ON, Canada; Division of Gastroenterology, Northwestern University, Chicago, IL, United States; Department of Medicine (Division of Gastroenterology) and Farncombe Family Digestive Health Research Institute, McMaster University, Hamilton, ON, Canada; Division of Gastroenterology and Hepatology, Departments of Medicine and Community Health Sciences, University of Calgary, Calgary, AB, Canada; Department of Medicine, Division of Gastroenterology, Western University, London, ON, Canada; Department of Internal Medicine III, Division of Gastroenterology and Hepatology, Medical University of Vienna, Vienna, Austria; Department of Medicine (Division of Gastroenterology) and Farncombe Family Digestive Health Research Institute, McMaster University, Hamilton, ON, Canada

**Keywords:** ulcerative colitis, corticosteroids, adverse events, clinical trials, remission

## Abstract

**Background and Aims:**

Adverse events (AEs) are frequently reported in clinical trials of advanced therapies for ulcerative colitis (UC). It remains uncertain whether patients receiving concomitant corticosteroids experience higher AE rates. This study aimed to determine whether corticosteroid use is associated with increased AEs in UC trials.

**Methods:**

This post-hoc analysis used participant-level data from several placebo-controlled trials (GEMINI-1, ULTRA-2, VARSITY, ACT-1, OCTAVE, and PURSUIT). The primary population included induction responders with or without baseline corticosteroid use. The primary outcome assessed the association between corticosteroid use and total AEs. Secondary outcomes included AE rates among those who achieved corticosteroid-free remission versus those who did not, and whether specific AEs were more common in corticosteroid users.

**Results:**

Among 2339 patients who achieved clinical response after induction, 1159 (49.5%) used corticosteroids at baseline. By 1 year, AE incidence was higher among corticosteroid users than non-users (75.2% vs. 67.6%, *P *< .001). Patients who achieved corticosteroid-free remission had fewer AEs than those who did not (67.4% vs. 77.5%, *P *< .001). Moderate AEs were more frequent among corticosteroid users. Common AEs included infections (in both groups) and liver abnormalities (more in corticosteroid users). Multivariable logistic regression confirmed baseline corticosteroid use as an independent AE risk factor [odds ratio (OR) 1.5, 95% CI 1.3–1.8, *P *= .002]. Ongoing corticosteroid use at 1 year was associated with even higher AE risk (OR 4.6, 95% CI 2.1–6.8, *P *< .001).

**Conclusion:**

Corticosteroid use is independently associated with increased AEs in UC clinical trials. Continued monitoring and strategies for corticosteroid tapering may reduce AE burden.

## Graphical abstract

**Graphical Abstract:**
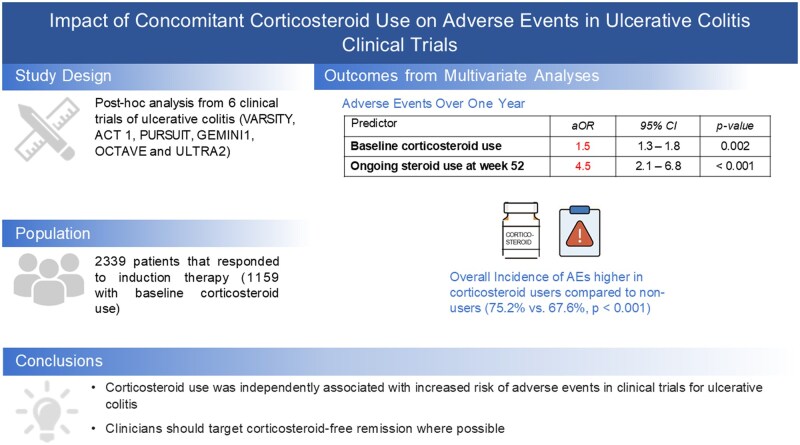

## 1. Background

Ulcerative colitis (UC) is a chronic inflammatory bowel disease characterized by mucosal inflammation that affects the colon.^1^ Clinically, UC is characterized by a relapsing and remitting course with symptoms including blood in the stool, diarrhea, urgency, incontinence, and abdominal pain; patients can also have systemic symptoms and/or extraintestinal manifestations. The etiology of UC is not known and therefore a curative therapy is unavailable. Corticosteroids remain widely used to control relapses of moderate and severe UC.^2^

Currently, several biologic and small molecule treatments have been approved and currently are used in the management of moderate-to-severe UC.^3^ Although such therapies have markedly improved the quality of life of UC patients, they are associated with various adverse events (AEs). Studies have shown an increased risk of AEs including opportunistic infections, malignancies, and miscellaneous complications such as injection/infusion reactions.^4–6^ Although risks have been observed mostly in patients using anti-tumor necrosis factor (anti-TNF) therapy, the risks of AEs are considered to be increased when using biologics in combination with corticosteroids or other immunosuppressive agents,^4–6^ with risk of opportunistic infections also linked to concomitant corticosteroids.^7^

There has been a notable absence of direct comparisons of AEs between patients with versus without concomitant corticosteroids while receiving advanced therapy for UC. Despite the increasing recognition of the challenges posed by corticosteroid dependence in UC management and the potential impact on AEs, a comprehensive analysis directly comparing these two distinct patient groups has not been undertaken. The primary objective of this study was to evaluate whether patients with UC who continue to use corticosteroids during treatment with advanced therapies within clinical trials have a higher rate of AEs compared to those not using corticosteroids and those who achieve clinical remission and discontinue steroids.

## 2. Methods

### 2.1. Study design

Participant-level data were obtained from multiple placebo-controlled clinical trials of moderate-to-severe UC. Data from GEMINI-1 (Clinicaltrials.gov: NCT00783718), ULTRA-2 (NCT00408629), OCTAVE-1 (NCT01465763), and VARSITY (NCT02497469) were obtained through Vivli (protocol #000010381), and by permission from Takeda, Pfizer, and AbbVie. In addition, data from ACT-1 (NCT00036439) and PURSUIT (NCT00488631) were obtained through the YODA (Yale Open Data Access #2024-0764) Project, and by permission from Janssen Inc. The Hamilton Integrated Research Ethics Board determined that local ethics review was unnecessary as data used in this analysis were previously collected and deidentified, and therefore no informed consent was required.

### 2.2. Exposures

The main exposure of interest was concomitant oral corticosteroid use. There were “treat-straight-through” studies (VARSITY, ACT-1, and ULTRA-2), and re-randomization studies (PURSUIT, GEMINI-1, and OCTAVE) included. To account for differences in study design, the cohort of patients analyzed were those who experienced a clinical response post-induction (and thus would have been eligible to enter a maintenance study), and outcomes among those using corticosteroids with clinical response were compared to participants not using corticosteroids with clinical response.

### 2.3. Variables

The main variable of interest was the number of AEs occurring by 1 year reported in the clinical trials along with the subtypes of AEs reported (eg, infection, colitis exacerbation, headache, upper respiratory tract infection, nasopharyngitis, anemia, and arthralgia), and their severity (mild, moderate, severe). Other variables that may have an association with the outcomes of interest were considered within the logistic regression analysis, including patient demographics (age, sex, race, weight, and current smoking), therapies used (advanced therapy used, concomitant immunomodulator), biochemical markers of disease [C-reactive protein (CRP), serum albumin], and disease activity (total Mayo score). All predictors were evaluated as binary or continuous variables.

### 2.4. Outcomes

The primary outcome of this study was to determine whether there was an association between concomitant corticosteroid use and total AEs by 1 year. A secondary outcome of interest was whether there was an association between concomitant corticosteroid use and infections during the clinical trials. Then, among baseline corticosteroid users, AEs were compared between those who were able to wean off corticosteroids and achieve clinical remission at 1 year vs those who could neither discontinue corticosteroids nor achieve remission at 1 year. Corticosteroid-free clinical remission was defined as the absence of corticosteroid use at the 1-year assessment with a total Mayo Score of ≤2 with no subscore >1.

### 2.5. Statistical analysis

Descriptive statistics were provided for each comparison. Continuous variables were presented as means (and SD) or medians (and IQR). Dichotomous variables were summarized as proportions or percentages. The patient pool of clinical responders was subdivided into those who used corticosteroids at baseline and those who did not. Then, among those who used corticosteroids at baseline, these participants were further stratified by those who achieved corticosteroid-free clinical remission at 1 year and those who were not able to wean off corticosteroids or achieve corticosteroid-free clinical remission at 1 year. The AEs were grouped based on severity as determined by local investigators and reported within the clinical trial: mild, moderate, and severe. The most frequent AEs from each group were also reported. Univariate analyses were conducted to evaluate the relationship between variables of interest and occurrence of AEs by 1 year. Subsequently, multivariate logistic regression using backward stepwise selection was performed and only those baseline variables with *P* < .05 on univariate analysis were considered for inclusion. Odds ratios (ORs) are presented with associated 95% CIs. Statistical significance was chosen to be at *P* < .05. Data were analyzed using Stata IC 15.0.

## 3. Results

### 3.1. Demographics

We included a total of 2339 patients who demonstrated clinical response to biologic or advanced therapy for UC. The cohort was divided into two groups: 1159 patients (49.6%) who were using corticosteroids at baseline and 1180 patients (50.4%) who were not. Baseline characteristics for both groups are presented in Table 1. The mean albumin level was significantly lower among those using corticosteroids compared to non-users (4.1 vs 4.3 g/dL, *P* < .001). A smaller proportion of those using corticosteroids had previously been treated with tumor necrosis factor (TNF) inhibitors (17.2% vs 20.5%, *P* = .034).

**Table 1.** Baseline characteristics: corticosteroid users vs non-users.

Variable	Corticosteroids at baseline	No corticosteroids at baseline	*P*-value
Total number of patients	1159 (49.5)	1180 (50.5)	
Age, years, mean (SD)	41.6 (14.5)	41.7 (13.5)	.624
Sex, male, *n* (%)	683 (59)	692 (58.6)	.922
Race, White, *n* (%)	839 (72.4)	814 (69.2)	.078
Weight, kg, mean (SD)	73.8 (16.4)	75.2 (14.2)	.949
Smoking, *n* (%)	63 (5.4)	45 (3.8)	.076
CRP, mg/L, median (IQR)	3.7 (9)	4.5 (9.4)	.155
Albumin, g/dL, mean (SD)	4.1 (0.4)	4.3 (0.4)	<.001*
Prior TNF inhibitor use, *n* (%)	197 (17.2)	242 (20.5)	<.034*
Concomitant immunomodulator, *n* (%)	225 (19.4)	270 (22.9)	.09
Total Mayo Score, mean (SD)	8.4 (1.6)	8.8 (1.6)	.264

Total number of patients at baseline is 2339. *P*-value was calculated using a chi-squared test for categorical variables and t-test for continuous variables.

* A *P*-value of <.05 is significant.

Among the 1159 patients who were using corticosteroids at baseline, 273 (23.5%) had corticosteroids weaned off and achieved clinical remission at 1 year, whereas 886 (76.5%) either remained corticosteroid-dependent or failed to achieve clinical remission at 1 year. Table 2 provides detailed baseline characteristics for these subgroups. A higher proportion of patients who achieved corticosteroid-free clinical remission were of white race (82.4% vs 88.1%, *P* = .019). Additionally, patients achieving corticosteroid-free remission had significantly lower baseline median CRP levels (2.9 mg/L vs 4.6 mg/L, *P *< .001) and higher mean albumin levels (4.3 g/dL vs 4.1 g/dL, *P *< .001). Patients who achieved corticosteroid-free clinical remission had significantly lower baseline mean total Mayo scores (8.0 vs 8.3, *P* = .007).

**Table 2.** Baseline characteristics: corticosteroid-free clinical remission vs corticosteroid-users or non-remission at 1 year.

Variable	Corticosteroid-free clinical remission at 1 year	Corticosteroid-users or non-remission at 1 year	*P*-value
Total number of patients	273	886	
Age, years, mean (SD)	40.9 (13.8)	41.2 (13.1)	.784
Sex, male, *n* (%)	164 (60)	513 (57.9)	.571
Race, White, *n* (%)	225 (82.4)	781 (88.1)	.019*
Weight, kg, mean (SD)	73.5 (16.3)	74.3 (17.1)	.322
Smoking, *n* (%)	23 (8.4)	95 (10.7)	.326
CRP, mg/L, median (IQR)	2.9 (4.9)	4.6 (10.3)	<.001*
Albumin, g/L, mean (SD)	4.3 (0.4)	4.1 (0.4)	<.001*
Prior TNF inhibitor use, *n* (%)	49 (17.8)	149 (16.8)	.732
Concomitant immunomodulator, *n* (%)	64 (23.5)	233 (26.3)	.387
Total Mayo Score, mean (SD)	8 (1.6)	8.3 (1.6)	.007*

Corticosteroid-free clinical remission at 1 year is defined as the absence of corticosteroid use at the 1-year assessment with a total Mayo Score of <2 with no subscore >1. Corticosteroid-users is defined as presence of corticosteroid use and non-remission is a total Mayo Score of >2 or a subscore >1 at the 1-year assessment. *P*-value was calculated using a chi squared test.

* A *P*-value of <.05 is significant.

### 3.2. Adverse events at 1 year for corticosteroid users vs non-corticosteroid users

The overall incidence of AEs at 1 year was significantly higher in patients who used corticosteroids at baseline compared to those who did not (75.2% vs 67.6%, *P *< .001). However, the infection rates were similar between the two groups (4.6% vs 4.9%, *P* = .77). Among patients using corticosteroids at baseline, the overall incidence of AEs was significantly lower in patients who achieved corticosteroid-free clinical remission at 1 year compared to those who did not wean corticosteroids or were not able to attain clinical remission (67.4% vs 77.5%, *P *< .001).

The severity of AEs was categorized as mild, moderate, or severe (Table 3). Patients receiving corticosteroids at baseline experienced a significantly higher proportion of moderate AEs compared to non-corticosteroid users (21.9% vs 15.5%, *P *< .001). In contrast, the incidence of mild AEs (44.8% in corticosteroid users vs 43.9% in non-corticosteroid users, *P* = .729) and severe AEs (3.9% in corticosteroid users vs 3.5% in non-corticosteroid users, *P* = .679) did not differ significantly between the groups.

**Table 3.** Adverse event severity at 1 year: corticosteroid users vs non-users.

Severity of AE	Corticosteroids at baseline (*n* = 1159)	No corticosteroids at baseline (*n* = 1180)	*P*-value corticosteroids vs non-corticosteroid
Mild (%)	519 (44.8)	519 (43.9)	.729
Moderate (%)	254 (21.9)	183 (15.5)	<0.001*
Severe (%)	45 (3.9)	41 (3.5)	.679

AE, adverse evens. *P*-value was calculated using a chi-squared test.

* A *P*-value of <.05 is significant.

The most common types of AEs differed between patients who used corticosteroids at baseline and those who did not (Table 4). Notably, liver function abnormalities emerged as the most prominent corticosteroid-associated AE, occurring at a numerically higher rate in corticosteroid users compared to non-users (9.9% vs 5.9%). Conversely, several AEs occurred more frequently numerically in the non-corticosteroid cohort, including upper respiratory tract infections (7.5% vs 3.8% in corticosteroid users), worsening colitis (8.1% vs 5.3% in corticosteroid users), headache (6.1% vs 4.5% in corticosteroid users), nasopharyngitis (5.4% vs 3.0% in corticosteroid users), and anemia (4.7% vs 3.0% in corticosteroid users).

**Table 4.** Most common adverse events by severity: corticosteroid users vs non-users.

Sum of types of AE	Corticosteroids at baseline (*n* = 1159)	No corticosteroids at baseline (*n* = 1180)
Colitis (%)	61 (5.3)	96 (8.1)
Infection (%)	70 (6)	72 (6.1)
Upper respiratory tract infection (%)	44 (3.8)	88 (7.5)
Headache (%)	52 (4.5)	72 (6.1)
Nasopharyngitis (%)	35 (3)	64 (5.4)
Anemia (%)	35 (3)	56 (4.7)
Arthralgia (%)	44 (3.8)	40 (3.4)
Nervous system disorder (%)	32 (2.8)	23 (1.9)
Skin and subcutaneous (%)	35 (3)	15 (1.3)
Liver function (%)	115 (9.9)	70 (5.9)

AE, adverse events; *n*, total number of patients.

### 3.3. Predictors of adverse events

To identify factors associated with AEs in patients with UC, we performed univariate and multivariate logistic regression analyses (Table 5). In the univariate analysis, several factors were significantly associated with the occurrence of AEs.

**Table 5.** Logistic regression analyses of adverse events at 1 year among clinical responders to advanced therapies.

	Univariate		Multivariate	
Predictor variable	Odds ratio (95% CI)	*P*-value	Adjusted odds ratio (95% CI)	*P*-value
Age	0.99 (0.98–1.02)	.79		
Sex	0.91 (0.53–1.58)	.75		
Race (White)	0.83 (0.47–1.44)	.49		
Smoking	0.63 (0.5–0.87)	.04*	N/A	.45
Weight	1 (0.99–1.01)	.45		
Albumin	1.14 (0.72–1.79)	.57		
CRP	0.74 (0.35–1.13)	.42		
Advanced therapy received (reference golimumab)				
Adalimumab	1.5 (1.4 - 2.9)	<.001*	2.3 (1.5–3.8)	<.001*
Vedolizumab	1.2 (0.65–2.36)	.52		
Infliximab	0.51 (0.26-0.99)	.048*	N/A	.36
Tofacitinib	1.9 (1.5–3.8)	<.001*	1.4 (1.2–1.5)	<.001*
Concomitant immunomodulator	0.9 (0.4 -1.9)	.78		
Baseline corticosteroids	1.1 (1.0-2.1)	.04*	1.5 (1.3–1.8)	.002*
Concomitant corticosteroids (week 52)	7.7 (5.6–10.6)	<.001*	4.6 (2.1–6.8)	<.001*
Total Mayo Score	1.02 (1–1.3)	.003*	1.7 (1.4–2.5)	.001*

* A *P*-value of <.05 is significant.

**Table 6.** Logistic regression analyses of adverse events at 1 year among severe ulcerative colitis patients with clinical response to advanced therapies (*n* = 600 with total Mayo score 9–12).

	Univariate		Multivariate	
Predictor variable	Odds ratio (95% CI)	*P*-value	Adjusted odds ratio (95% CI)	*P*-value
Age	0.9 (0.96–1.02)	.67		
Sex	1.3 (0.67–2.65)	.41		
Race (White)	1.9 (0.92–4.1)	.08		
Smoking	0.4 (0.11–0.77)	.55		
Weight	1 (0.97–1.03)	.91		
Albumin	0.9 (0.4–1.02)	.13		
CRP	0.4 (0.15–1.4)	.44		
Advanced therapy received (reference golimumab)				
Adalimumab	1.2 (1.1–1.8)	<.001*	1.6 (1.2–3.5)	<.001*
Vedolizumab	1.3 (0.45–3.5)	.67		
Infliximab	1.8 (0.67–5.5)	.27		
Tofacitinib	0.6 (0.2–0.8)	**<.01** *	1.9 (1.1–2.2)	<.001*
Concomitant immunomodulator	0.7 (0.3–2.2)	.59		
Baseline corticosteroids	1.7 (1.3–2.3)	<.001*	1.2 (1.1–1.4)	.01*
Concomitant corticosteroids (week 52)	2.2 (1.5–3.1)	<.001*	3.1 (2.2–5.6)	<.001*

* A *P*-value of <.05 is significant.

In the multivariate model after backward selection, four factors remained independently associated with AEs: advanced therapy used [adalimumab (OR 2.3, 95% CI 1.5–3.8, *P *< .001) and tofacitinib (OR 1.4, 95% CI 1.2–1.5, *P *< .001) compared to golimumab], baseline corticosteroid use (OR 1.5, 95% CI 1.3–1.8, *P* = .002), concomitant corticosteroid use at week 52 (OR 4.6, 95% CI 2.1–6.8, *P *< .001), and total Mayo score (OR 1.7, 95% CI 1.4–2.5, *P* = .001). Notably, smoking and infliximab vs golimumab, which were significant in univariate analysis, did not maintain statistical significance in the multivariate model (*P* = .45 and *P* = .36, respectively). A subgroup analysis was performed among patients with severe UC only (total Mayo score 9–12) (Table 6). There was a total of 600 patients included within this subgroup (241 with total Mayo score of 9, 190 with total Mayo score of 10, 128 with total Mayo score of 11, and 41 with total Mayo score of 12). Within this severe subgroup, baseline corticosteroid use (OR 1.2, 95% CI 1.1–1.4, *P* = .01) and concomitant corticosteroid use at week 52 (OR 3.1, 95% CI 2.2–5.6, *P *< .001) remained predictive of AEs over 1 year, These findings suggest that specific advanced therapies, corticosteroid use (particularly long-term), and disease severity are independent risk factors for AEs in patients with UC.

## 4. Discussion

Although corticosteroids are an important short-term treatment option for patients with moderate to severe UC, repeated or prolonged exposure are associated with harms. To our knowledge, this is the largest study that has investigated the relationship between oral corticosteroids and the occurrence of AEs in patients with moderate to severe UC undergoing advanced treatments with a comparable reference arm of patients not using corticosteroids. Our results indicated that patients who used corticosteroids at baseline exhibited a higher rate of AEs (75.2% vs 67.6%, *P *< .001) compared to non-corticosteroid users. On the other hand, among patients who were initially on corticosteroids, those who were able to wean and achieved corticosteroid-free clinical remission at 1 year experienced a lower overall number of AEs compared to those who could neither discontinue corticosteroids nor achieve remission (67.4% vs 77.5%, *P *< .001).

Our findings align with the established evidence that corticosteroids increase the risk of AEs and discontinuing them could reduce the risk of AEs. A comprehensive meta-analysis by Lichtenstein et al. demonstrated that corticosteroid use is associated with a 2.5-fold increased risk of serious infections when combined with biologic therapy in IBD patients.^8^ Similarly, a recent study by Fidder et al. showed that concomitant corticosteroid use increased the risk of AEs in UC patients undergoing anti-TNF therapy by 2.7-fold.^9^^,^^10^ This is further supported by our multivariate analysis, which identified both baseline corticosteroid use (OR 1.5, 95% CI 1.3–1.8, *P = *.002) and concomitant corticosteroid use at week 52 (OR 4.6, 95% CI 2.1–6.8, *P *< .001) as independent risk factors for AEs, with the latter demonstrating a particularly strong association. The substantially higher odds ratio for continued corticosteroid use at week 52 compared to baseline corticosteroid use suggests a duration-dependent effect, consistent with findings from Waljee et al. who demonstrated that cumulative corticosteroid exposure significantly increases AE risk in IBD patients.^11^

Liver function abnormalities emerged as the most prominent corticosteroid-associated AE in our study, occurring at a substantially higher rate in corticosteroid users compared to non-users (9.9% vs 5.9%). This finding is consistent with recent literature documenting the hepatic effects of corticosteroids. A 2021 review by Chalasani et al. reported that approximately 30% of IBD patients experience abnormal liver function tests during their disease course, with drug-induced liver injury being a significant contributor.^12^ Corticosteroids can induce hepatic steatosis through metabolic alterations and may potentiate hepatotoxicity when combined with other medications commonly used in UC management.^13^ The mechanism likely involves glucocorticoid-induced upregulation of lipogenic enzymes and inhibition of beta-oxidation of fatty acids, leading to hepatic fat accumulation.^14^ Additionally, the potential for drug–drug interactions between corticosteroids and biologics or immunomodulators may further contribute to liver function abnormalities, as suggested by a recent pharmacokinetic study by Verstockt et al.^15^ The higher prevalence of liver function abnormalities in our corticosteroid-using cohort underscores the importance of regular liver function monitoring in these patients.

Susceptibility to infection is of great concern to both physicians and patients taking immunosuppressive drugs. Interestingly, in our study, infection rates were similar between those using corticosteroids at baseline compared to those who did not (4.6% vs 4.9%, *P* = .77). This finding contrasts with some previous studies that have reported increased infection risks with corticosteroid use. A possible explanation for the similar infection rates seen at baseline could be related to the lower doses of corticosteroids used in our study cohort. Among the trials included in our analysis, the highest dose patients were taking was 20 mg of prednisone daily, which is below the threshold associated with significantly increased infection risk (typically >20 mg/day) as reported by Toruner et al.^7^ Additionally, a recent meta-analysis by Singh et al. suggested that the combination of corticosteroids and biologics at lower corticosteroid doses may not substantially increase infection risk compared to biologic monotherapy.^16^ This dose-dependent effect on infection risk is further supported by Brassard et al., who found that the risk of serious infections increases significantly only with prednisone-equivalent doses exceeding 20 mg daily.^17^ Even in non-IBD populations, use of lower doses of prednisone (≤10 mg/day) was not associated with a higher risk of infectious complications.^18^

Disease severity also emerged as an independent risk factor for AEs in our multivariate analysis (OR 1.7, 95% CI 1.4–2.5, *P* = .001 for total Mayo score). This finding has important clinical implications, as it suggests that the relationship between treatment and AEs is confounded by disease activity. Patients with more severe disease may experience more AEs not only due to more intensive treatment but also due to the disease process itself. This concept is supported by a recent study by Ananthakrishnan et al., which demonstrated that inflammatory burden, independent of treatment, is associated with increased risk of adverse outcomes in IBD patients.^19^^,^^20^ The relationship between disease severity and AEs highlights the complex interplay between disease activity, treatment intensity, and safety outcomes in UC management, emphasizing the need for a balanced approach that considers both efficacy and safety when making treatment decisions.

In clinical practice, corticosteroids are often used to manage flares and are required in many jurisdictions before advanced therapies are reimbursed. The findings of our study suggest that clinicians should aim to wean corticosteroids off as early as possible and target a corticosteroid-free remission state. British guidelines recommend that steroids be weaned off by 8 weeks, and real-world data have suggested that the majority of IBD patients can achieve steroid discontinuation by 8 weeks with no increase in flare-ups or hospitalizations.^21^^,^^22^

The strength of our study resides in the utilization of participant-level data from multiple placebo-controlled clinical trials, providing a robust dataset for comprehensive analysis of a sizable and diverse patient population. The inclusion of multiple clinical trials enhances the generalizability of the findings, and the consistency in data collection protocols across these trials strengthens the reliability of the results. Furthermore, our study specifically examined the impact of achieving corticosteroid-free remission on AE rates, an outcome that has been increasingly recognized as clinically meaningful but remains underreported in many trials.^23^^,^^24^ The use of multivariate analysis to identify independent predictors of AEs while controlling for potential confounders represents another methodological strength of our study.

The limitations of our study include the post-hoc nature of the analysis using data from multiple clinical trials, which introduces inherent biases. Patients using corticosteroids at baseline are likely inherently different from those who are not and may have been “sicker” patients, which warranted use of corticosteroids at outset. This potential confounding by indication is partially addressed by our multivariate analysis, which adjusted for disease severity, but residual confounding cannot be excluded. Additionally, some patients may have been using other concomitant therapies, such as immunomodulators, which could also potentially affect the risk of AEs, although notably we did not observe a higher rate of immunomodulator use among those using corticosteroids compared to those who were not.

Moreover, there were differences in corticosteroid-weaning regimens across the studies, which may have influenced the rates of AEs observed. A recent systematic review by George et al. highlighted the heterogeneity in corticosteroid tapering protocols across clinical trials in UC, which can impact both efficacy and safety outcomes.^24^ Differences in baseline corticosteroid doses and duration of use during the trial may influence rate of AEs but was not considered within this study. Finally, there were no data collected regarding the duration of corticosteroid use before entering the trial, which could increase the risk of AEs in those with longer cumulative corticosteroid use. This limitation is important to acknowledge, as cumulative corticosteroid exposure has been associated with dose-dependent increases in AEs in recent real-world studies.^25^ Future studies should prospectively collect data on pre-trial corticosteroid exposure and standardize corticosteroid tapering protocols to better elucidate the relationship between corticosteroid use patterns and AE risk.

In conclusion, our study confirms that AEs are higher among patients using corticosteroids within clinical trials of UC, which aligns with conventional understanding regarding corticosteroid use. Achieving corticosteroid-free clinical remission offers many potential benefits, including an overall reduction in AEs as observed in this study. The differential safety profiles observed with various biologic agents and the complex relationship between disease severity, treatment modality, and AEs underscore the importance of personalized treatment approaches in UC management. Clinicians should carefully weigh the benefits of corticosteroid therapy against the increased risk of AEs and strive to achieve corticosteroid-free remission whenever possible. Further research is needed to explore the impact of cumulative corticosteroid dose, optimal weaning regimens, and long-term safety outcomes in patients transitioning from corticosteroids to advanced therapies.

## Data Availability

Data can be made available upon request through VIVLI/YODA.
